# Evaluation of a targeted, theory-informed implementation intervention designed to increase uptake of emergency management recommendations regarding adult patients with mild traumatic brain injury: results of the NET cluster randomised trial

**DOI:** 10.1186/s13012-018-0841-7

**Published:** 2019-01-17

**Authors:** Marije Bosch, Joanne E. McKenzie, Jennie L. Ponsford, Simon Turner, Marisa Chau, Emma J. Tavender, Jonathan C. Knott, Russell L. Gruen, Jill J. Francis, Sue E. Brennan, Andrew Pearce, Denise A. O’Connor, Duncan Mortimer, Jeremy M. Grimshaw, Jeffrey V. Rosenfeld, Susanne Meares, Tracy Smyth, Susan Michie, Sally E. Green

**Affiliations:** 1 Department of Surgery, Monash University, Melbourne, Australia; 2 National Trauma Research Institute, Alfred Hospital and Monash University, Melbourne, Australia; 3 Faculty of Economics and Business, University of Groningen, Groningen, The Netherlands; 4 School of Public Health and Preventive Medicine, Monash University, Melbourne, Australia; 5 Monash-Epworth Rehabilitation Research Centre, Epworth Hospital, Melbourne, Australia; 6 School of Psychological Sciences, Monash University, Melbourne, Australia; 7 Melbourne Medical School, The University of Melbourne, Melbourne, Australia; 8 Department of Emergency Medicine, Royal Melbourne Hospital, Melbourne, Australia; 9 Lee Kong Chian School of Medicine, Nanyang Technological University, Singapore, Singapore; 10 School of Health Sciences, City University of London, London, UK; 11 MedSTAR Emergency Medical Retrieval Service, Adelaide, Australia; 12 Royal Adelaide Hospital Emergency Department, Adelaide, Australia; 13 Centre for Health Economics, Monash Business School, Monash University, Melbourne, Australia; 14 Clinical Epidemiology Program, Ottawa Hospital Research Institute, Ottawa, ON Canada; 15 Department of Medicine, University of Ottawa, Ottawa, ON Canada; 16 Department of Neurosurgery, The Alfred Hospital, Melbourne, Australia; 17 Department of Psychology, Macquarie University, Sydney, Australia; 18 Emergency Department, Westmead Hospital, Sydney, Australia; 19 Department of Clinical, Educational and Health Psychology, University College London, London, UK

**Keywords:** Mild traumatic brain injury, Cluster trial, Effectiveness, Emergency department, Implementation science, Clinical practice guideline, Evidence-based practice

## Abstract

**Background:**

Evidence-based guidelines for management of mild traumatic brain injury (mTBI) in the emergency department (ED) are now widely available; however, clinical practice remains inconsistent with these guidelines. A targeted, theory-informed implementation intervention (Neurotrauma Evidence Translation (NET) intervention) was designed to increase the uptake of three clinical practice recommendations regarding the management of patients who present to Australian EDs with mild head injuries. The intervention involved local stakeholder meetings, identification and training of nursing and medical local opinion leaders, train-the-trainer workshops and standardised education materials and interactive workshops delivered by the opinion leaders to others within their EDs during a 3 month period. This paper reports on the effects of this intervention.

**Methods:**

EDs (clusters) were allocated to receive either access to a clinical practice guideline (control) or the implementation intervention, using minimisation, a method that allocates clusters to groups using an algorithm to minimise differences in predefined factors between the groups. We measured clinical practice outcomes at the patient level using chart audit. The primary outcome was appropriate screening for post-traumatic amnesia (PTA) using a validated tool until a perfect score was achieved (indicating absence of acute cognitive impairment) before the patient was discharged home. Secondary outcomes included appropriate CT scanning and the provision of written patient information upon discharge. Patient health outcomes (anxiety, primary outcome: Hospital Anxiety and Depression Scale) were also assessed using follow-up telephone interviews. Outcomes were assessed by independent auditors and interviewers, blinded to group allocation.

**Results:**

Fourteen EDs were allocated to the intervention and 17 to the control condition; 1943 patients were included in the chart audit. At 2 months follow-up, patients attending intervention EDs (*n* = 893) compared with control EDs (*n* = 1050) were more likely to have been appropriately assessed for PTA (adjusted odds ratio (OR) 20.1, 95%CI 6.8 to 59.3; adjusted absolute risk difference (ARD) 14%, 95%CI 8 to 19). The odds of compliance with recommendations for CT scanning and provision of written patient discharge information were small (OR 1.2, 95%CI 0.8 to 1.6; ARD 3.2, 95%CI − 3.7 to 10 and OR 1.2, 95%CI 0.8 to 1.8; ARD 3.1, 95%CI − 3.0 to 9.3 respectively).

A total of 343 patients at ten interventions and 14 control sites participated in follow-up interviews at 4.3 to 10.7 months post-ED presentation. The intervention had a small effect on anxiety levels (adjusted mean difference − 0.52, 95%CI − 1.34 to 0.30; scale 0–21, with higher scores indicating greater anxiety).

**Conclusions:**

Our intervention was effective in improving the uptake of the PTA recommendation; however, it did not appreciably increase the uptake of the other two practice recommendations. Improved screening for PTA may be clinically important as it leads to appropriate periods of observation prior to safe discharge. The estimated intervention effect on anxiety was of limited clinical significance. We were not able to compare characteristics of EDs who declined trial participation with those of participating sites, which may limit the generalizability of the results.

**Trial registration:**

Australian New Zealand Clinical Trials Registry (ACTRN12612001286831), date registered 12 December 2012.

**Electronic supplementary material:**

The online version of this article (10.1186/s13012-018-0841-7) contains supplementary material, which is available to authorized users.

## Introduction

Traumatic brain injury, caused by external forces such as sports, falls or accidents, is a frequent presentation to emergency departments (EDs) worldwide [1]. The vast majority (80 to 90% depending on the definition) are classified as ‘mild’ severity. People with mild traumatic brain injury (mTBI) are usually managed in the ED and discharged within hours [2]. The challenge for ED clinicians is to identify which patients presenting with a head injury require further management and which patients can safely be sent home [3]. While the majority of people suffering mTBI will make a full recovery within a few weeks or months, approximately 15–25% will go on to subjectively report post-concussion symptoms such as ongoing headaches, memory and concentration problems, and sleep difficulties [4–6]. A small minority (approximately 1%) have underlying intracranial haemorrhage and deteriorate quickly, requiring neurosurgical intervention [7].

Several high quality evidence-based clinical practice guidelines are available to guide the care of patients who present to the ED with mTBI [8]. Three key clinical practice recommendations from these guidelines determined as important in an Australian setting [8, 9] are (1) post-traumatic amnesia (PTA) should be prospectively assessed in ED using a validated tool; (2) guideline-developed criteria or clinical decision rules should be used to determine the appropriate use and timing of computed tomography (CT) imaging; (3) verbal and written patient information consisting of advice, education and reassurance should be provided upon discharge from the ED. Despite the availability of guidelines, research undertaken within Australia and internationally has shown that care is often inconsistent with these recommendations [10–14] (see Additional file 1 (Table 1) for further information on the three key recommendations, their relevance to managing this patient group and the evidence underpinning the recommendations).

This gap between guideline recommendations and actual practice is not unique to mTBI, with similar difference identified in many clinical disciplines. We know that the dissemination of guidelines alone is seldom sufficient to change practice [15, 16] and more active strategies aiming to bring about practice change are needed. These implementation strategies may be more effective if they are underpinned by theories of behaviour change and consider the context and determinants of practice (both barriers to and enhancers of the recommended practice) [17, 18]. Implementation studies incorporating the explicit use of theories in the processes of designing and evaluating targeted interventions [18] has been recommended in emergency settings [19, 20], as relatively few implementation studies have been conducted compared with other settings.

As part of a program of research aiming to improve outcomes for patients with mTBI (the Neurotrauma Evidence Translation (NET) program) [21], we developed an implementation intervention to increase the uptake of the three key clinical practice recommendations. To maximise the likelihood of the intervention’s effectiveness, our intervention was informed by evidence and theories of change [22] and designed to target the identified determinants of practice (e.g. address the barriers and enhance the enablers) [23, 24]. The NET-Trial [25] aimed to test the effectiveness of this implementation intervention, compared with the dissemination of a guideline on the management of mTBI patients presenting to ED [26].

### Aim and objectives

Our primary objective was to establish whether the intervention increased the percentage of patients for whom a prospective measure of PTA using a validated tool was performed in the ED until a perfect score was achieved (indicating absence of acute cognitive impairment) or the patient was transferred or admitted.

Secondary objectives included establishing whether the intervention increased the percentage of patients for whom two other assessment methods of PTA were performed, for whom CT scanning was appropriately performed; who received written patient information upon discharge from the ED; and who received appropriate care according to outcomes measuring the implementation of multiple (composite) recommendations. In addition, we hypothesised that the provision of appropriate patient information [27] upon discharge from the ED would reduce anxiety and the number of self-reported symptoms. We also investigated the effects of our intervention on post-accident functioning (return to normal activities including work and health-related quality of life (HRQoL)) and head injury-related re-presentations. Finally, we aimed to assess the cost-effectiveness of the intervention and we conducted a process evaluation to aid the interpretation of the trial results. In this paper, we report the effects of the intervention on clinical practice and patient outcomes.

## Methods

A protocol for this study has been published (Additional file 1) [25] and a brief overview of the methods follows. We describe deviations from planned methods (Additional file 2) and provide further detail of methods that had not been fully developed at the time of publication of the protocol. A completed CONSORT for cluster randomised trial reporting checklist, which indicates the sections of the paper where each reporting item is addressed, is available in Additional file 3. The trial was registered in the Australian New Zealand Clinical Trials Registry on 12 December 2012 (ACTRN12612001286831).

### Ethics statement

The trial protocol was approved by the Alfred Health Human Research Ethics Committee (approval Number 398/12). Following recruitment, additional local ethics and research governance procedures were completed for each site. Details of consent and confidentiality procedures are available in the study protocol (Additional file 1).

### Study design

The study design was a cluster randomised trial. Each cluster included an ED with its medical and nursing clinicians and the patients treated with mTBI. A cluster randomised design was primarily chosen because the intervention was targeted at ED staff. Two levels of participation in the study were offered, which we term NET and NET-Plus. In NET, clinical practice outcomes, but not patient outcomes, were measured, while in NET-Plus, both were measured.

### Recruitment of EDs and inclusion/exclusion criteria

Recruitment of EDs occurred between February 2013 and October 2013. We approached EDs listed in the Australasian Society for Emergency Medicine ED Directory list of 24-h Australian EDs [28]. EDs were contacted in batches. All non-responding sites were followed up by email and phone. Exclusion criteria were (1) specialised hospitals not routinely treating adults with mTBI; (2) no CT scanner on site; (3) risk of contamination due to two EDs having the same ED Director, or senior influential clinicians working across sites (in which case only one ED was allowed to participate); and (4) sites having involvement in the pilot and/or development of the intervention. Hospitals were included if the ED director provided consent to enter the study (either NET or NET-Plus) on behalf of their staff by returning a completed consent form. Details of the ED recruitment process and consent procedures are outlined in the protocol (Additional file 1).

### Identification of patients and inclusion/exclusion criteria

A retrospective chart audit of the ED medical records was conducted to identify eligible patients (see Additional file 4 for details on this process). Patients meeting the following criteria were included: (1) aged 18 or older, (2) presented to the ED within 24 h of injury, (3) sustained an acute blunt head trauma, and (4) had a GCS score of 14 or 15 at presentation [26]. Patients meeting the following criteria were excluded: (1) penetrating injuries and (2) non-traumatic brain-injury such as stroke. Two additional exclusion criteria were added: (3) patient left the ED before being seen or discharged themselves, and (4) the patient medical record was missing, with reasons outlined in Additional file 2. A waiver was granted to undertake the process of retrieving records of patients meeting our inclusion criteria without patient consent.

### Recruitment of patients for follow-up and inclusion/exclusion criteria (NET-Plus only)

In hospitals which chose to participate in the NET-Plus study component, eligible patients identified from chart audit were contacted by telephone by an ED staff member and invited to participate in a follow-up telephone interview by psychologists experienced in interviewing individuals with brain trauma. Additional exclusion criteria for the NET-Plus component included (1) not being able to participate in a telephone interview (e.g. we were unable to support patients with hearing-impairments or provide translation services for patients who spoke languages other than English), (2) cognitive impairment from intellectual disability and/or neurological syndrome, and (3) severe substance use disorder and/or major psychiatric disorder requiring hospitalisation. Informed consent from patients to pass their contact details to the NET research team was first sought by the ED staff member. Following consent to share contact details, an information sheet was posted to the patient, which provided a 2-week opt-out option. After 2 weeks without opt-out, consent to participate was presumed. Prior to conducting the interview, the psychologists re-checked inclusion criteria. Patients were able to opt-out of the interview at any time.

### Randomisation and allocation concealment

EDs were allocated to intervention or control groups using minimisation, a method that allocates clusters to groups using an algorithm to minimise differences in predefined factors between the groups [29]. Minimisation was implemented in the package *minim* [30]. Pure minimisation is completely deterministic; however, the algorithm we implemented included a random element. The allocation of EDs to intervention groups was undertaken externally to preclude any potential influence in the allocation by trial staff, study investigators, or study participants (i.e. ED directors). A statistician independent of the study implemented the minimisation in two batches. The statistician was only provided with ED identification codes and minimisation variables and was instructed to randomly sort the order in which the EDs would be entered into the minimisation package. The minimisation factors included (1) existence of a protocol for appropriate PTA assessment in mTBI patients, (2) size (annual presentation rate 2012), (3) rurality, and (4) level of participation (NET or NET-Plus).

### Blinding

Due to the nature of the intervention, it was not possible to blind ED staff members to group allocation. To limit the possibility of selection and detection bias, chart auditors were independent of the hospital and blinded to ED group allocation. In addition, medical records staff who retrieved the records, patient interviewers and the statistician who performed the analyses were blinded to group allocation.

### Intervention

The method of development of the intervention has been reported elsewhere [22]. In brief, prior to designing the intervention, we conducted interviews to identify the clinical and organisational factors that may influence the implementation of the three recommended practices [23, 24]. The content of the intervention was designed to target the important factors identified through the interviews. Both the interviews and the intervention design process were guided by two theoretical frameworks in a complementary manner [22]. The first of these, the Theoretical Domains Framework (TDF), is grounded in psychological theories of clinical behaviour change [31]. The second, the Model of Diffusion of Innovations in Service Organisations, was developed from an organisational perspective [32]. Next, intervention components were identified and operationalised. Behaviour change techniques that were most likely to bring about change for each clinical practice were identified, using sources that link techniques to the theoretical domains of the TDF [33–35]. In addition, the literature was consulted to identify intervention components that might be effective in targeting or taking into account organisational factors that were identified through the interviews [32, 36–39]. Finally, evidence on the effectiveness of interventions designed to improve healthcare delivery [40, 41] and information derived from the interviews regarding practicalities and feasibility of proposed intervention components was considered. Table 1 presents an overview of the delivery of intervention components, and Additional file 5 provides further details on the content and rationale for including each component.

**Table 1:** Delivery of the intervention

Intervention components	
Intervention and control group	
1. An electronic/printed copy of *Initial management of closed head injury in adults* guideline [26]. Intervention sites received an electronic copy of the guideline at the Train-the-Trainer workshop. Control group departments received their copy in between the first and second Train-the-Trainer event (July 2014). When control sites asked for guidance on what to do with the guideline, they were instructed to do what they would normally do if they became aware of a guideline relevant to their practices.	
2. Data collection reminder sticker/flag in system and education around the importance of documenting information for mTBI patients to optimise data collection.	
Intervention group only	
3. One hour face-to-face multidisciplinary stakeholder meeting in each participating ED with key stakeholders (both clinical and organisational/change management) and senior NET clinicians and researcher to create buy-in at ‘organisational’ level for the changes by discussing the key recommendations and underlying evidence; discussing intervention components and how to overcome anticipated barriers for their implementation.	
4. Identification of multidisciplinary local opinion leader team (medical and nursing) via key-informant method [69] (ED Directors were provided with a description of the types and characteristics of people suited to the role (Additional file 5)	
5. One day train the trainer interactive workshop, led by content experts and senior NET clinicians, attended by the nursing and medical opinion leaders, consisting of information provision and skills training both in relation to the key recommendations as well as in relation to their role in the study	
6. Following the Train-the-Trainer workshop, opinion leaders were asked to provide training to their staff members over a 3 month period of time. Opinion leaders were provided with power-point presentations with standardised text and other training materials such as case descriptions and pre-recorded demonstration sessions.	
7. Provision of relevant tools and materials (e.g. PTA screening tools, CT-head rules [26] and patient information booklets [27] translated into five languages that are commonly spoken in Australia)	

### Control

Control EDs received the guideline and data collection reminders only (components 1 and 2, Table 1). They were offered the full intervention following the conclusion of the trial.

### Outcomes

The clinical practice and patient outcomes are described in Table 2. These represent a subset of all outcomes measured in the trial; effects of the intervention for the other outcomes (proxy measures of clinical practice and predictors of clinical practice (Additional file 1)) will be reported in a separate publication. Clinical practice outcomes include those which measure implementation of single and multiple (composite) recommendations.

**Table 2:** Clinical practice and patient outcomes

Outcome	Definition of outcome measure	Potential range of responses/interpretation of scales	Outcome assessment period/timing	Data collection method
Clinical practice outcomes (measured on all patients)				
Outcomes measuring the implementation of single clinical recommendations				
Appropriate post-traumatic amnesia screening (PTA)^*^	Prospective assessment of PTA appropriately undertaken, where appropriately undertaken was defined as using a validated tool, until a perfect score was achieved (indicating absence of acute cognitive impairment) before the patient was discharged home (or the patient was admitted or transferred)	Yes or no	Retrospectively on 2 month period post-intervention	Chart audit
PTA screening-tool	The administration of the validated tool was completed at least once	Yes or no	Retrospectively on 2 month period post-intervention	Chart audit
Memory-clinical assessment	Clinicians had made an assessment of PTA using questions in their clinical assessment	Yes or no	Retrospectively on 2 month period post-intervention	Chart audit
CT scan-clinical criteria (CT)	A CT scan was provided in the presence of a risk factor that justified the scan (age 65 or older; GCS < 15; amnesia; suspected skull fracture; vomiting and coagulopathy) [25] (assessed in the cohort of patients for whom risk criteria were recorded only)	Yes or no	Retrospectively on 2 month period post-intervention	Chart audit
CT scan (all) ^&^	A CT scan was provided or not	Yes or no	Retrospectively on 2 month period post-intervention	Chart audit
Provision of written patient information (INFO)	Written information was provided to the patient on discharge home from the ED	Yes or no	Retrospectively on 2 month period post-intervention	Chart audit
Outcomes measuring the implementation of composite recommendations				
Safe discharge based on PTA and INFO	Safe discharge based on whether the patient received appropriate care for the two practices PTA and INFO (assessed for all patients)	Yes or no	Retrospectively on 2 month period post-intervention	Chart audit
Safe discharge based on PTA, CT, and INFO	Safe discharge based on whether the patient received appropriate care for all of the three clinical practices PTA, CT, and INFO (assessed in the cohort of patients for whom risk criteria were recorded)	Yes or no	Retrospectively on 2 month period post-intervention	Chart audit
Patient outcomes (measured on NET-Plus only patients)				
Anxiety	All 7 anxiety items from the Hospital Anxiety and Depression Scale [70, 71]. Each item is rated on a 4-point scale from 0 to 3, with 3 indicating higher symptom frequency. The scores were summed across the 7 items to create an anxiety score	Score between 0 and 21, with higher scores indicating greater anxiety. (A score > 7 indicates clinically significant anxiety)	3 to 5-month post-discharge^#^	Patient telephone interview
Post-concussion symptoms (RPQ-13)	13 item Rivermead scale (RPQ-13) [72]. Each item measured on a 5-point scale from 0 (not experienced) to 4 (severe problem). The scores were summed across the 13 items to create the RPQ-13 score	Score between 0 and 52, higher scores indicate greater severity of post-concussion symptoms.	3 to 5-month post-discharge	Patient telephone interview
Post-concussion symptoms (RPQ-3)^&^	3 item Rivermead scale (RPQ-3) [72]. Each item measured on a 5-point scale from 0 (not experienced) to 4 (severe problem). The scores were summed across the 3 items to create the RPQ-3 score.	Score between 0 and 12, higher scores indicate greater severity of post-concussion symptoms	3 to 5 month post-discharge	Patient telephone interview
Not returned to normal activities	Based on three items: (1) whether the patient was doing the same working hours as before the incident (if applicable), (2) whether the patient was studying the same hours as before the incident (if applicable), and (3) whether the patient was back to their other normal activities such as gardening, buying groceries, visiting friends or family, or other leisure activities. Each item was coded ‘No’ or ‘Yes’. The three items were then combined; if one of these items was scored ‘no’, the patient was considered to have not returned to normal activities.	No or Yes. ‘Yes’ means the patient has not returned to normal activities.	3 to 5-month post-discharge	Patient telephone interview
Health-related quality of life (SF6D)	SF6D index scores, derived from 12-item short form health survey (SF-12) [73, 74].	Scores between 0.350 (the ‘pits’) and 1.000 (‘full health’), higher scores indicate higher HRQoL.	3 to 5-month post-discharge	Patient telephone interview
mTBI-related re-presentation^$^	The patient re-presented within a month of the initial presentation for an mTBI-related reason	Yes or no	Retrospectively on 2 month period post-intervention	Chart audit

^*^Primary outcome

^#^Patient interviews took place between 4.3 and 10.7 months post-presentation. Reasons for the difference between planned and actual patient follow-up are outlined in Additional file 2

^$^Chart audit data

^&^Outcome additional to trial protocol. Reasons for inclusion outlined in Additional file 2

### Data collection processes

We collected baseline cluster characteristics including type of hospital (public or private), rurality and whether the site had a protocol for appropriate PTA assessment in mTBI patients via telephone with ED Directors or delegates. Clinical practice outcomes, patient characteristics and re-presentations were measured through retrospective chart audit. The follow-up period was 2 months in length and began post-delivery of the last intervention component at each site (ranging between October 2014 and February 2015). During follow-up, notices were implemented at all hospitals to remind staff of the importance of recording decisions in medical records for mTBI patients. Patient outcomes were collected via telephone interview.

### Data quality assurance

Chart auditors were trained with the aim of maximising consistency in applying inclusion and exclusion criteria and collecting data from medical records. The auditors received a data collection manual with instructions regarding data entry in the web-based database (including a data dictionary), and weekly phone meetings were held to discuss questions. In addition, data were downloaded twice during each hospital audit and data checks were run to identify inconsistencies and errors, which were discussed with chart auditors the same day to enable correction of data entries.

Researchers conducting patient interviews received training and supervision to ensure standardised delivery of interview questions. Patient outcome data were entered directly into the web-based database.

The database was designed to minimise errors through real-time checks.

### Sample size

To detect an absolute increase of 20% in the rate of appropriate PTA screening (equivalent to an odds ratio of 3.9, log odds 1.3) (assuming a control group rate of 10%, an intra-cluster correlation (ICC) of 0.18, coefficient of variation of cluster size of 0.47, an average of 30 patient participants per ED, and a two-sided 5% significance level) with approximately 80% power, we required 15 EDs per intervention group. A total of 30 EDs would provide 900 patient participants for whom ED staff management would be assessed. Allowing for 10% attrition, we planned to initially recruit 34 EDs. Rationale and empirical support for the sample size parameters and justification for the target difference we wished to detect between groups is provided in the trial protocol (Additional file 1). The sample size calculations were undertaken using the module clustersampsi [42], implemented in the statistical package Stata (StataCorp LP, USA) [43].

### Effectiveness analyses

The effectiveness of the intervention for the clinical practice and patient outcomes was estimated with marginal modelling using generalised estimating equations (GEEs). These models appropriately account for the correlation of responses of individuals within EDs. An exchangeable correlation structure was specified, whereby responses from the same ED were assumed to be equally correlated [44]. We used robust variance estimation which yields valid standard errors even if the within-cluster correlation has been incorrectly specified [45, 46]. For binary outcomes, a logit link was used. For continuous outcomes, model specification tests were undertaken to determine the probability distribution and link function.

All models included adjustment for minimisation factors (see ‘Randomisation and allocation concealment’ section) and additionally (unless otherwise noted) for pre-specified confounders that included patients’ age, sex, and whether they presented after hours. All confounders were included in the models even when no baseline imbalance existed. Our primary effectiveness analysis was the model (as described above) that estimated the intervention effect on the primary outcome, appropriate PTA screening.

Estimates of intervention effect from the models with binary outcomes yielded odds ratios. To aid interpretability, we also provided estimates of absolute risk differences [47], computed from marginal probabilities estimated from the fitted logistic models [44]. Analysis additions and deviations from the protocol are outlined in Additional file 2.

## Results

### Participation of EDs

**Fig. 1:**
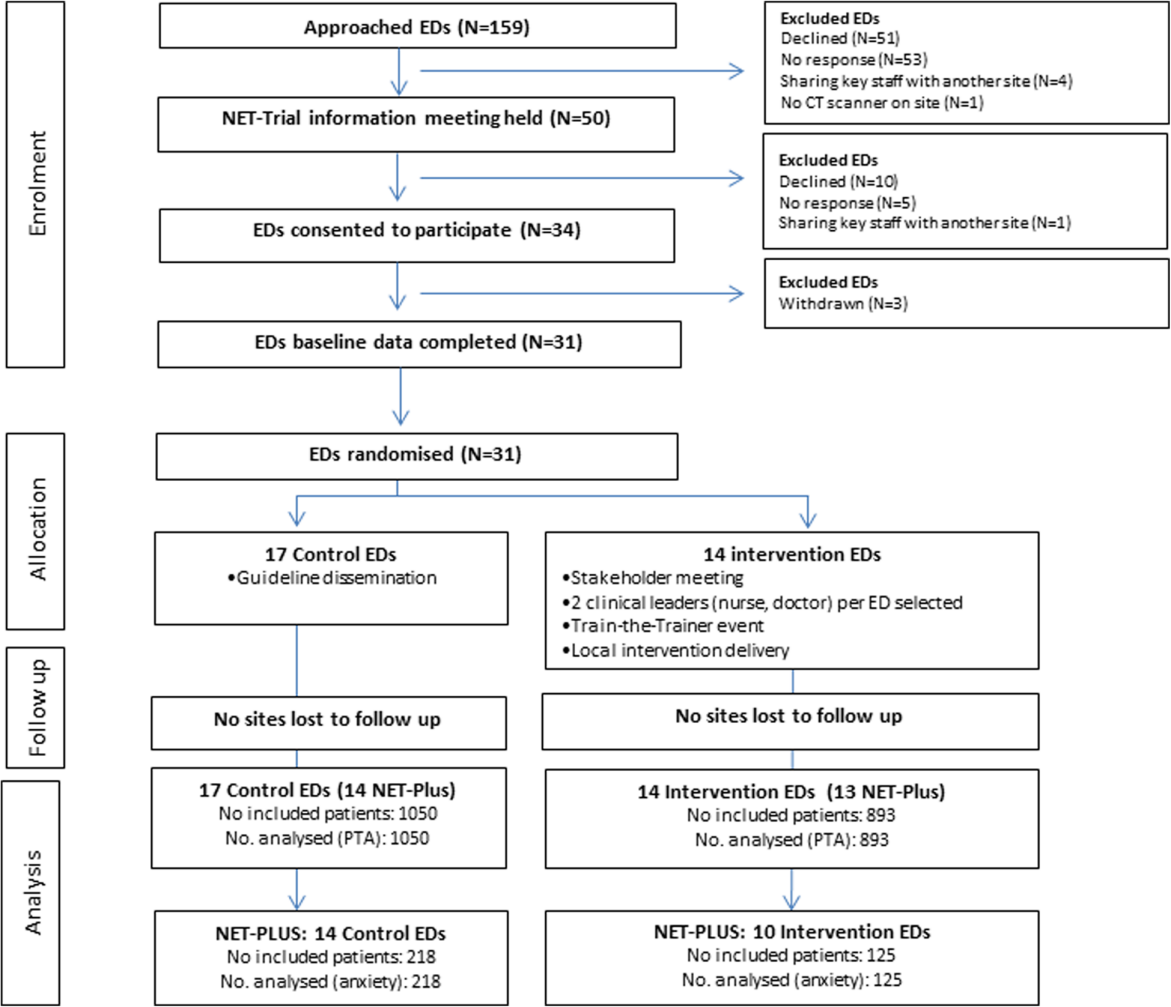
Participant flow diagram

Fifty-three expression of interest forms were received, and 50 information meetings held. Subsequently, 34 ED Directors (or delegates) provided written consent to participate in the trial. Three sites declined after or during completion of ethics documentation, which left 31 sites for completion of baseline characteristics and randomisation. Fourteen sites were allocated through minimisation to the intervention group and 17 sites to the control group. Twenty-seven sites consented to participate in NET-Plus, three of which (all intervention sites) did not recruit patients for follow-up interviews. Figure 1 shows the flow of sites through the trial.

### Baseline characteristics of EDs and clinicians

Participating EDs were primarily public hospitals, and the majority were based in urban areas. Less than a quarter of EDs had a protocol for appropriate PTA assessment. Groups were well balanced across the demographic measures (Table 3).

**Table 3:** Baseline demographic characteristics of EDs

ED structural characteristics	Control (no. of clusters = 17)	Intervention (no. of clusters = 14)
	*N* (%) or mean (SD) [median (IQR)]*	*N* (%) or mean (SD) [median (IQR)]*
Hospital type (private)	1 (6%)	1 (7%)
Hospital type (public)	16 (94%)	13 (93%)
Trauma unit	3 (18%)	4 (29%)
Short stay unit	13 (76%)	10 (71%)
Existence of protocol for mTBI	4 (24%)	3 (21%)
NET-Plus	14 (82%)	13 (93%)
Rurality (regional)	7 (41%)	5 (36%)
Annual presentation rate 2012	44,710 (22593) [42,495 (34,313 to 46,690)]	41,255 (16512) [41,574 (27,075 to 55,667)]

*Statistics presented are number (percent) or mean (standard deviation) [median (interquartile range)]

### Demographic and clinical characteristics of included patients

#### NET-Trial patients

A total of 1943 patients were identified from medical records for inclusion in the study (Table 4, column 2 and 3). The demographic and clinical characteristics of the patients were similar between groups, although intervention patients had more frequently experienced other injuries and had a higher mean age.

**Table 4:** Patient characteristics

Patient characteristics	NET control^1^	NET intervention^2^	NET-Plus control ^3^	NET-Plus intervention ^4^
	*N* (%^5^)/mean (SD)	*N* (%^5^)/mean (SD)	*N* (%^5^)/mean (SD)	*N* (%^5^)/mean (SD)
Age	50.9 (23.65)	54.2 (24.93)	53.5 (20.59)	55.2 (21.17)
Sex (male)	476 (45%)	390 (44%)	105 (48%)	51 (41%)
After hours presentation	748 (71%)	653 (73%)	149 (68%)	90 (72%)
Initial GCS 15	961 (92%)	768 (86%)	213 (98%)	115 (92%)
Initial GCS 14	89 (8%)	125 (14%)	5 (2%)	10 (8%)
Mechanism of injury				
Incidental fall	492 (47%)	481 (54%)	113 (52%)	65 (52%)
Road traffic	58 (6%)	51 (6%)	11 (5%)	10 (8%)
Violence / assault	250 (24%)	163 (18%)	36 (17%)	15 (12%)
Sport	62 (6%)	55 (6%)	17 (8%)	11 (9%)
Others	179 (17%)	137 (15%)	41 (19%)	24 (19%)
Unclear/not reported	9 (0.9%)	6 (0.7%)	0 (0.0%)	0 (0.0%)
Presence other injuries (outside head)	508 (48%)	516 (58%)	105 (48%)	63 (50%)
Alcohol/illicit drug involvement	237 (23%)	206 (23%)	28 (13%)	15 (12%)
Pre-existing coagulopathy or anti-coagulant or anti-platelet drugs	175 (17%)	165 (18%)	37 (17%)	17 (14%)
Known previous neurological condition	202 (19%)	191 (21%)	26 (12%)	13 (10%)
Known neurosurgery	14 (1.3%)	18 (2.0%)	2 (0.9%)	3 (2.4%)
Scalp laceration	532 (51%)	464 (52%)	130 (60%)	67 (54%)
Scalp haematoma	400 (38%)	372 (42%)	79 (36%)	42 (34%)
Clinical suspicion of skull fracture	51 (4.9%)	57 (6%)	8 (3.7%)	8 (6%)
Loss of consciousness	186 (18%)	155 (17%)	50 (23%)	18 (14%)
Vomiting	56 (5%)	49 (5%)	12 (6%)	4 (3.2%)
Headache	259 (25%)	231 (26%)	44 (20%)	37 (30%)
Post traumatic seizure	3 (0.3%)	6 (0.7%)	0 (0.0%)	2 (1.6%)
Focal neurological deficit	21 (2.0%)	13 (1.5%)	5 (2.3%)	3 (2.4%)

^1^Number of patients = 1050; numbers of clusters = 17

^2^Number of patients = 893; number of clusters = 14

^3^Number of patients = 218; number of clusters = 14

^4^Number of patients = 125; number of clusters = 10

^5^Percentages of less than 5% are given to one decimal place

#### NET-Plus patients

In total, 343 patients participated in the patient follow-up study (Table 4, column 4 and 5). Compared to the intervention group, the control group included a slightly higher proportion of males, more with loss of consciousness and fewer with headache recorded in their notes.

### Effects of the intervention on clinical practice outcomes

#### Clinical practice outcomes

##### Outcomes measuring the implementation of single clinical recommendations

Patients from EDs in the intervention group compared to those in the control group were more likely to have been appropriately assessed for PTA (primary outcome; adjusted OR 20.1, 95%CI 6.8 to 59.3, *p* <  0.001; which converts to an adjusted absolute risk difference (ARD) of 14%, 95%CI 8 to 19, Table 5). However, the percentage of patients who were screened appropriately in both groups was small (1% and 13% in the control and intervention groups respectively). Patients from intervention EDs were more likely to have had at least one administration of the validated PTA tool (PTA screening-tool; adjusted OR 19.7, 95%CI 6.6 to 58.1, *p* <  0.001) and to have had an assessment of PTA where the clinician used clinical questions (but no tool) (memory-clinical assessment; adjusted OR 1.6, 95%CI 1.2 to 2.1, *p* = 0.001).

**Table 5:** Estimated effects of the intervention on clinical practice outcomes

	NET control^1^		NET intervention^2^						
	No. of patients	No. of (%)	No. of patients	No. of (%)	Adj. ORs^**§§^	95%CI	*p* value	Adj. ARD %^^^	95%CI
Outcomes measuring the implementation of single clinical recommendations									
Appropriate post-traumatic amnesia screening (PTA)^*^	1050	12 (1.1)	893	117 (13)	20.1	(6.8, 59.3)	< 0.001	14	(8, 19)
PTA screening-tool	1050	15 (1.4)	893	152 (17)	19.7	(6.6, 58.1)	< 0.001	17	(11, 23)
Memory-clinical assessment	1050	272 (26)	893	303 (34)	1.6	(1.2, 2.1)	0.001	9.5	(4.0, 15.1)
CT scan-clinical criteria (CT)^§^	494	337 (68)	491	352 (72)	1.2	(0.8, 1.6)	0.375	3.2	(− 3.7, 10.0)
CT scan (all)	1050	458 (44)	893	446 (50)	1.2	(0.9, 1.6)	0.142	4.5	(− 1.5, 10.5)
Provision of written patient information (INFO)	944	175 (19)	785	160 (20)	1.2	(0.8, 1.8)	0.302	3.1	(− 3.0, 9.3)
Outcomes measuring the implementation of composite recommendations									
Safe discharge based on PTA and INFO	944	2 (0.2)	785	45 (6)	27.6	(6.9, 110.5)	< 0.001	5.8	(2.7, 8.9)
Safe discharge based on PTA, CT, and INFO^§§§^	413	0 (0)	402	14 (3.5)	1.8	(1.1, 3.0)	0.022	3.5	(1.0, 6.0)

^1^Number of clusters = 17

^2^Number of clusters = 14

ORs = odds ratios

^*^Primary outcome

^§^Criteria that justify a scan are age 65 or older; GCS < 15, amnesia, suspected skull fracture, vomiting and coagulopathy. Only the subset of patients who have these symptoms noted in the medical records are included in the analysis

^**^Adjusted odds ratios estimated from marginal logistic regression models using generalised estimating equations with an exchangeable correlation structure (unless otherwise noted) and robust variance estimation to allow for clustering of responses within EDs

^§§^All models (unless otherwise noted) adjusted for the minimisation factors and pre-specified confounders (see ‘Effectiveness analyses’ section)

^§§§^For this outcome, because there were no safe discharges in the control group, a cluster-level analysis was undertaken resulting in a ratio of geometric mean proportions. Details available in Additional file 2

^^^ARD calculated from marginal probabilities [75]. Confidence intervals for the metric were obtained by a pairwise comparison of margins after fitting a GEE model using Stata [43] allowing for clustering of observations within EDs

The difference between groups in the odds of compliance with recommendations for CT scanning were small and not statistically significant (Table 5). For both outcomes (CT scan-clinical criteria, and CT scan (all)), the odds were 1.2 times higher in the intervention compared with the control group (95%CI 0.8 to 1.6 and 0.9 to 1.6 respectively). In both groups, around 70% of patients for whom risk factors were noted in the medical record received a scan that was justified by those symptoms.

Similarly, the difference between groups in the odds of compliance with the recommendation for the provision of written patient discharge information was small (adjusted OR 1.2, 95%CI 0.8 to 1.8). In both groups, only around 20% of patients received written patient information upon discharge from the ED.

##### Outcomes measuring the implementation of multiple (composite) recommendations

Patients from EDs randomised to the intervention compared to the control group were more likely to have had safe discharge, both based on PTA and INFO (whole cohort; adjusted OR 27.6, 95%CI 6.9 to 110.5, *p* <  0.001) and based on PTA, CT and INFO (subset of cohort; adjusted OR 1.8, 95%CI 1.1 to 3.0, *p* = 0.022). In both groups, however, percentages of patients who received appropriate care according to our composite indicators of clinical practice were very low. This was caused by the low baseline rates for PTA and INFO predominantly.

### Effects of the intervention on patient outcomes

In total, 343 patients were interviewed at follow-up (Tables 4 and 6). The mean number of days between their ED presentation and the follow-up interview was 210 days (SD 38.5 days; IQR 181–239). The intervention had a small effect on anxiety, with the observed difference being − 0.52 (95%CI − 1.34 to 0.30, *p* = 0.216, Table 6) in favour of the intervention. Rates of post-concussion symptoms were low in both groups, and the intervention had only a small effect on RPQ-13 scores, which were 1.15 (95%CI − 2.77 to 0.48) lower for intervention patients, and RPQ-3 scores, which were 1.10 (95%CI − 0.48 to 0.28) lower in intervention patients. The percentage of patients who had not returned to normal activities was 19% and 13% in the control and intervention groups respectively (adjusted OR 0.67, 95%CI 0.28 to 1.61; which converts to an adjusted ARD of − 4.6% (95%CI − 16.2 to 7.0). There was uncertainty of the impact of the intervention on SF6D HRQoL scores which were 0.03 (95%CI 0.00 to 0.06) higher for those in the intervention group. The confidence interval included both important [48, 49] and trivial differences. The odds of re-presentation for those in the intervention group were nearly twice those in the control group (adjusted OR 1.92, 95%CI 1.08 to 3.40, *p* = 0.026, which converts to an adjusted ARD of 2.1%, 95%CI 0.3 to 3.8), a small difference of uncertain clinical significance.

**Table 6:** Effects of the intervention on patient outcomes

Patient interview responses		NET-Plus control		NET-Plus intervention				
	Value range	No. of patients/clusters	Mean (SD)/*N* (%)	No. of patients/clusters	Mean (SD)/*N* (%)	Adjusted effect^^^	95%CI	*p* value
Anxiety^1^	0 to 21	218/14	4.3 (4.01)	125/10	3.4 (3.58)	MD − 0.52^^^^	(− 1.34, 0.30)	0.216
Post-concussion symptoms (RPQ-13)^2^	0 to 52	218/14	6.7 (8.65)	125/10	4.7 (5.52)	MD − 1.15^^^^	(−2.77, 0.48)	0.167
Post-concussion symptoms (RPQ-3)^3^	0 to 12	218/14	1.16 (1.83)	125/10	0.90 (1.44)	MD − 1.10^^^^	(−0.48, 0.28)	0.611
Not returned to normal activities^4^	0 or 1	218/14	41 (19%)	126/10	16 (13%)	OR 0.67^^^^^	(0.28, 1.61)	0.368
SF6D HRQoL^5^	0.35 to 1	208/14	0.78 (0.14)	123/10	0.80 (0.13)	MD 0.03^^^^	(0.00, 0.06)	0.053
mTBI-related re-presentation^6^	0 or 1	1050/17	25 (2.4%)	893/14	39 (4.4%)	OR 1.92^^^^^^	(1.08, 3.40)	0.026

^1^Anxiety measured using the anxiety items in the Hospital Anxiety and Depression Scale giving a score between 0 and 21, higher scores indicate higher levels of anxiety and a score > 7 indicates clinically significant anxiety

^2^Post-concussion symptoms measured using the 13-item Rivermead scale (RPQ-13) giving a score between 0 and 52, higher scores indicate greater severity of post-concussion symptoms

^3^Post-concussion symptoms measured using the 3-item Rivermead scale (RPQ-3) giving a score between 0 and 12, higher scores indicate greater severity of post-concussion symptoms

^4^Whether or not a patient returned to normal activities was indicated by the patient answering no to any of the following: “Are you doing the same working hours as before the incident?” “Are you studying the same hours as before the incident?” “Are you back to (your) other normal activities such as gardening, buying groceries, visiting friends or family, or other leisure activities etc.?”

^5^SF6D index scores, derived from SF12v2 raw data using weights from Brazier and Roberts [76]

^6^Chart audit data

^^^Adjusted effects from models fitted using generalised estimating equations with an exchangeable correlation structure (unless otherwise noted) and robust variance estimation to allow for clustering within hospitals. Models adjusted for the design strata and pre-specified confounders (see ‘Effectiveness analyses’ section). Adjusted effects are adjusted mean differences (denoted MD) or adjusted odds ratios (denoted OR)

^^^^Modelled with independent within-group correlation structure. See ‘Effectiveness analyses’ section for details

^^^^^Adjusted ARD − 4.6% (95%CI − 16.2%, 7.0%)

^^^^^^Adjusted ARD 2.1% (95%CI 0.3%, 3.8%)

### Sensitivity analyses: effect estimates from models adjusting for minimisation criteria only

For clinical practice outcomes, the effect estimates from models in which there was only adjustment for the minimisation factors (see Additional file 2) were not appreciably different compared with the models that in addition adjusted for pre-specified confounders (Additional file 6). The exception to this was the primary outcome ‘appropriate PTA screening’, where the OR of 20.1 (95%CI 6.8 to 59.3) from the full model reduced to 15.6 (95%CI 5.0 to 48.8) for the model that only included the minimisation factors. This difference was influenced by the imbalance in age at baseline, where patients in the intervention group were on average older, and appropriate PTA screening was more likely to occur in younger patients. For patient outcomes, no meaningful differences were observed between the effect estimates obtained from models with and without adjustment for pre-specified confounders.

### Intra-cluster correlations (ICCs) for the primary outcomes

The ICC for our primary clinical practice outcome (appropriate PTA screening) was 0.12 (95%CI 0.06 to 0.19). However, estimates of ICCs differed for the two groups, with the ICC in the intervention group (0.06 (95%CI 0.00 to 0.11)) being smaller than the control (0.20 (95%CI 0.08 to 0.32)), potentially suggesting that clinical practice for PTA screening may have become more consistent across intervention EDs (Additional file 6—ICCs for clinical practice outcomes). The difference in prevalence rates between the groups may also provide a part explanation for the differences in estimated ICCs [50]. The ICC for our primary patient outcome (anxiety) was 0.02 (95%CI 0.01 to 0.07; Additional file 6).

## Discussion

We conducted a trial of a targeted, theory-informed implementation intervention to increase the uptake of clinical practice recommendations for the management of patients presenting to Australian EDs with mTBI. Results suggest that our intervention improved management, increasing the percentage of patients being appropriately assessed for PTA and of ‘safe discharge’ (based on both composite scores). The observed improvement in our composite measures mainly reflects the improvement in PTA as the intervention did not appreciably increase the uptake of the other two practice recommendations. The impact of the intervention on patient outcomes was generally in favour of the intervention group, but estimated effects were small and of limited clinical significance. Anxiety levels at follow-up in both groups were low (intervention mean 3.4, SD 3.58; control mean 4.3, SD 4.01), and the intervention had only a small effect in favour of intervention patients (adjusted mean difference − 0.52, 95%CI − 1.34 to 0.30; scale 0–21). The latter is perhaps unsurprising since our intervention did not improve the provision of patient information, which was the mechanism by which we hypothesised anxiety would improve.

While the observed effect for our primary outcome was smaller than the 20% difference in absolute improvement, we powered our trial to detect (Additional file 1), the effect was in fact larger than that observed in many trials testing similar interventions [41]. Further, the confidence bounds suggest that the true intervention effect could plausibly be as small as an 8% improvement, or as large as a 19% improvement, with the latter magnitude consistent with that which we set out to detect. This improvement means that more patients received care in concordance with best clinical practice; they had a record of PTA duration (which is important for diagnosis and management) [51], and fewer patients were sent home in unsafe conditions (i.e., while still experiencing acute but temporary cognitive impairment) [52, 53]. The intervention effect was somewhat larger for the ‘PTA screening tool’. This demonstrates that PTA screening was started but not maintained until the patient had a perfect score before the patient was determined safe for discharge. EDs may find it difficult to repeat assessment under time and resource pressure. However, this is a crucial aspect of PTA assessment.

As is the case with every new intervention, ultimately, health service providers and fundholders would need to decide what size of improvement would be important enough in their setting to justify any increase in costs associated with adopting the intervention. The economic evaluation that was conducted alongside this trial considered the trade-off between the net costs of the implementation intervention and improvements in clinical practice and health outcomes. The authors conclude that, as delivered in the trial, the balance of costs and outcomes from the implementation intervention is unlikely to be acceptable to providers and fundholders. Full results and further reflections on this can be found in Mortimer et al. [54].

While very few EDs in both groups were screening for PTA, there may have been less room for improvement for CT scanning. CT rates were 44% in the control group at follow-up. Comparison between studies is complicated due to differences in definitions, methods, study population and the fact that the criteria that justified a scan in our study differ from published studies. However, this potentially indicates there was only modest room for improvement, as the percentage of CT head scans in this patient group that would have been required by applying various head rules (calculated by dividing the number of mTBI patients in whom the decision rule was positive divided by the total number of mTBI patients) has been estimated to range roughly between 50 and 70% ([55–57], although lower (42% [57], 43% [58]) and higher (79 and 96%) [57] rates have also been reported). This does not apply however to the provision of written discharge information, with no more than 20% of patient records in both groups including documentation of patients receiving written materials, although these latter rates may have been influenced by incomplete recording (see study strengths and limitations).

Many factors may explain why the intervention was effective in increasing the uptake of PTA screening, but not the clinical recommendations related to appropriate CT scanning and provision of patient information on discharge. For example, it may have been the case that the content of the intervention (e.g. the components we selected) did not address all identified barriers for these practices. It could also be that the intervention was not implemented as intended (e.g. content surrounding PTA assessment received more attention in the local training workshops provided within the EDs by the opinion leaders as it was a new or less familiar practice). Our process evaluation will provide insight on potential explanations.

### What this study adds to the literature

To our knowledge, few studies have evaluated theory-informed, targeted interventions in an ED setting. A cluster trial that included 12 matched pairs of community hospitals [59] concluded that their implementation intervention failed to significantly increase alteplase use in patients with ischaemic stroke. Although their intervention addressed local barriers in each intervention site, the intervention was similar to that of the NET trial in that it was designed to alter systems and behaviour at an institutional level and individual staff level, focusing on change in the ED setting. The intervention was based on behaviour change theory and adapted from previous experience in the development of alteplase delivery systems.

In terms of contributing to the body of knowledge relating to the implementation of guideline recommendations, the NET trial will inform research examining the effectiveness of organisational and professional interventions in emergency practice settings in increasing uptake of research evidence [60], as well as—in any setting—the effectiveness of multi-faceted interventions versus single-component interventions [61], the effectiveness of targeted versus non-targeted interventions [18] and of theory-informed interventions versus other interventions [18] in increasing the uptake of recommendations.

### Study strengths and limitations

This study has limitations. First, the majority of EDs invited to participate in the trial declined participation. This may therefore limit the generalisability of the results since the characteristics of the non-participating EDs may have differed, and these characteristics may influence the effectiveness of the intervention.

As we were conducting a retrospective audit to select patients for inclusion in the trial, we relied on discharge coding in the medical record systems to identify patients. Previous research has shown coding in TBI populations is likely to be incomplete and/or inaccurate [62–64] particularly when other injuries were involved. Therefore, it is likely we have missed mTBI patients in our audits. Where possible, we included text searches in triage notes using head injury-related terms to identify patients with non-head injury-related codes. The proportion of sites where this was not possible was similar across groups. The comparability of the patient characteristics demonstrates that the identification processes were implemented similarly by group.

Although previous studies have indicated incomplete information in patient records [65], we collected data from medical records retrospectively. Therefore, our trial outcomes were dependent on the recording practices and clinical information available in the medical records. Previous studies have noted this may particularly be an issue for practices such as providing advice [66, 67]. Indeed, in over 55% of patients, no information on receipt of patient information at discharge was recorded. For patients who had records of written patient information upon discharge in their files, we were unable to determine whether the information provided was in fact the intervention booklet [27]. Several alternative patient information sheets are available for EDs, and these may not include information such as reassurance and the importance of gradual return to activities. In addition, due to missing information, we were not able to study appropriate denial of CT head, as this would require recorded evidence in the notes of the absence of all possible criteria justifying a scan.

We intended to improve recording in the medical record systems by implementing a data collection reminder in all participating EDs. In addition, we asked study coordinators to remind their staff of the importance of including full information in the clinical record during the trial catchment period. However, it is unlikely that this would have led to differences in incomplete reporting between groups.

The duration of the follow-up of the study may also have been a limitation. We collected chart audit data over the 2 months directly following a 3-month local intervention delivery period in each site. It could be the case that this period was too short for the intervention to be fully embedded in routine practice. Conversely, it is also conceivable that the observed intervention effect fades out over time. Therefore, it would have been informative to study sustainability by including a later time point for repeat chart audit [68]. This was not feasible within the timeframe of the trial. In addition, although originally planned to take place at 3 to 5 months post-injury, delays in recruitment of participants by ED staff meant that clinical follow-up in the NET-Plus trial did not take place until an average of 7 months post-injury and there was fairly wide range in time post-injury at which participants were followed up (control mean 209.1 days, SD 35.9 and intervention mean 212.7, SD 42.6). This may have impacted on rates of reporting of symptoms. Also, the intervention may have had an early clinically important effect on patient outcomes that we could not assess.

This study also has some clear strengths, such as the process used to minimise selection bias in the allocation of EDs to the intervention groups through our implementation of the minimisation method. This included (i) using a minimisation algorithm that had a random element so that the allocation was not fully deterministic, (ii) having a statistician independent of the trial implement the process using batches of EDs, and (iii) randomly sorting the order in which EDs were entered into the minimisation program. Additional strengths are that we used a systematic process to design the intervention in order to maximise the likelihood of effectiveness, that we included objective measures of practice and that we included a process evaluation as well as an economic evaluation. In addition, we used independent chart auditors and only one chart auditor (who also was an experienced ED nurse) selected patient records for retrieval by medical records departments of participating sites. Several measures were in place to assure consistent data entry between chart auditors, such as training, phone meetings on a weekly basis to discuss any questions, circulation of decisions taken and real-time data downloads and checks based on algorithms. Finally, the EDs we recruited were distributed across the country.

## Conclusions

We report the results of a large, nationwide trial of a targeted, theory-informed implementation intervention in emergency care settings where, to date, relatively few trials have been carried out. The intervention had an important impact on appropriate PTA assessment, but did not have an appreciable impact on appropriate CT scanning and written patient information on discharge at 2 months follow-up. Further, the impact of the intervention on patient outcomes was either clinically uncertain or not clinically important. Future evaluations may focus on modifying the developed intervention to bring about larger improvement and longevity of the effects of the intervention.

## Additional files

Additional file 1: Trial protocol. (PDF 1390 kb)

Additional file 2: Deviations from study protocol. (PDF 282 kb)

Additional file 3: CONSORT checklist. (PDF 301 kb)

Additional file 4: Patient identification protocol. (PDF 205 kb)

Additional file 5: Overview of NET-Trial intervention and rationale for selection of components. (PDF 447 kb)

Additional file 6: Extra tables. ICCs and estimated effects of the intervention on clinical practice and patient outcomes, adjusting for the minimisation criteria only. (PDF 303 kb)

## Abbreviations

CT: Computed tomography
ED: Emergency department
GCS: Glasgow Coma Scale
HRQoL: Health-related quality of life
ICC: Intra-cluster correlation
INFO: Provision of written patient information upon discharge
mTBI: Mild traumatic brain injury
NET: Neurotrauma Evidence Translation
PTA: Post-traumatic amnesia
RPQ: Rivermead Post-Concussion Symptoms Questionnaire
SF-12: 12-item Short Form Health Survey
SF6D: Short-Form Six-Dimension
