# Supplementary material for: Evaluation of a targeted, theory-informed implementation intervention designed to increase uptake of emergency management recommendations regarding adult patients with mild traumatic brain injury: results of the NET cluster randomised trial
Source: Implement Sci. 2019 Jan 17;14:4. doi: 10.1186/s13012-018-0841-7 (PMC6337860; doi:10.1186/s13012-018-0841-7)
Supplement: Supplementary file 2 — Deviations from study protocol. (PDF 282 kb) [file 13012_2018_841_MOESM2_ESM.pdf]

Additional file 2: Deviations from study protocol

	Plan as per study protocol	Deviation form protocol *	Reason for deviation from study protocol
Identification of patients and inclusion/exclusion criteria	Our pre-defined exclusion criteria were: 1) penetrating injuries, and 2) non-traumatic TBI such as caused by stroke.	Two additional exclusion criteria were added: 3) patient left the ED before being seen or discharged themselves, and 4) the patient medical record was missing.	These criteria were added to prevent missing data for these reasons (in both cases we were not able to assess the management of a particular patient).
Clinical practice outcomes (patient level)<br>*CT scanning outcome*	The protocol only contained the outcome measure 'CT scan-clinical criteria'. This outcome measured whether a CT scan was provided in the presence of a risk factor that justified the scan, in the cohort of patients for whom risk criteria were recorded.	We made a post-hoc decision to include an additional outcome, 'CT scan (all)', which measured whether a CT scan was provided or not (in all patients).	This additional outcome was included so that we would have a measure of the impact of the intervention on CT scanning across all patients (and not just a subset of patients for whom risk criteria were recorded). Given hospitals were randomly assigned to intervention groups, we would expect that the case-mix of participants would be similar across groups, and therefore that comparing CT-scan rates for all patients would yield an unbiased estimate of intervention effect.
Patient outcomes	We planned to perform patient follow-up interviews three to five months post presentation.	Patient interviews took place between 4.3 and 10.7 months post-presentation.	This change was due to logistical issues. It took longer than expected to recruit local ED staff who were tasked with contacting patients to seek their consent. The ED staff also took longer than we had planned to complete these consent procedures.
Patient outcomes	Post-concussion symptoms was measured using the 13-item Rivermead [1], which represent the cluster of 'late' symptoms	We decided post examination of the data to add the three item Rivermead (RPQ-3), measuring 'early symptoms; symptoms experiences directly after the event' [1] as an additional secondary outcome.	As we planned to measure long-term post-concussion symptoms (three to five months post-presentation), we decided to use the RPQ-13, as we expected late symptoms to have more impact on psychosocial functioning then those reported immediately after injury [1]. However, we found headaches (which is part of the RPQ-3) to be the third most common symptom in our sample, so we decided to also report on the cluster of early symptoms (RPQ-3) in addition to the cluster of late symptoms (RPQ-13).
Analyses section additional analyses	Models will include adjustment for minimisation factors and pre-specified potential confounding variables	In addition to presenting effect estimates from models that adjust for the minimisation factors and pre-specified potential confounding variables, as a sensitivity analysis we also present effect estimates from models that adjust for the minimisation factors only.	Comparison between intervention effects estimated from both models allows examination of the degree of agreement..
Analyses section additional analyses	We had not specified what we would do when the estimated ICC was negative	For models where the estimated ICC was negative, we refitted the model with an independent correlation structure, which assumes an ICC of zero	Fitting the GEE with an independent correlation structure yields conservative estimates of standard errors and is consistent with recommendations and practices of others [2, 3]
Use of alternative statistical methods	Use of GEEs with a logit link for binary outcomes	For the composite measure safe discharge based on appropriate care for all three clinical practices (PTA, CT, and INFO), we undertook a cluster level analysis where we calculated the proportion of patients who were safely discharged for each cluster and from this calculated i) a	For the composite measure safe there were no safe discharges in the control group, precluding the use of the logistic model because of the phenomenon of separation.

		risk difference, as the (unweighted) difference in the average proportions between groups, and ii) a ratio of geometric mean proportions, as the exponential of the difference in the average log proportions between groups [4]. We obtained confidence intervals and p-values using statistical inference based on the t-distribution. For the ratio of geometric means, we dealt with zero safe discharges in clusters by adding 0.5 to the number of safe discharges in all clusters before dividing by the number of patients [4].	
Analyses section additional analyses	We had not specified what statistical method to estimate the ICC	We calculated ICCs using analysis of variance (ANOVA). Confidence intervals for the ICCs were bootstrapped using the combination of bootstrap and loneway commands in Stata 14 [5]. We allowed for the clustering of observations within EDs. Bias corrected 95% confidence intervals were calculated using 1000 replicates. ICCs were calculated for all EDs, and by intervention group separately.	

*: All statistical analyses were undertaken using Stata version 14 [5]. GEE = generalised estimating equations. ICC = intra-cluster correlation.

1. King NS, Crawford S, Wenden FJ, Moss NE, Wade DT. The Rivermead Post Concussion Symptoms Questionnaire: a measure of symptoms commonly experienced after head injury and its reliability. Journal of neurology 1995, 242(9):587-592.
2. Ukoumunne OC, Gulliford MC, Chinn S, Sterne JA, Burney PG. Methods for evaluating area-wide and organisation-based interventions in health and health care: a systematic review. Health Technol Assess 1999, 3(5):iii-92.
3. Eldridge S, Kerry S, Torgerson DJ. Bias in identifying and recruiting participants in cluster randomised trials: what can be done? BMJ 2009, 339:b4006.
4. Hayes RJ, Moulton LH. Analysis Based on Cluster-level Summaries In: Cluster Randomised Trials. edn.: Chapman and Hall/CRC; 2009: 163-197.
5. StataCorp: Stata Statistical Software: Release 14. College Station, TX: StataCorp LP 2015.
