# Supplementary material for: Evaluation of a targeted, theory-informed implementation intervention designed to increase uptake of emergency management recommendations regarding adult patients with mild traumatic brain injury: results of the NET cluster randomised trial
Source: Implement Sci. 2019 Jan 17;14:4. doi: 10.1186/s13012-018-0841-7 (PMC6337860; doi:10.1186/s13012-018-0841-7)
Supplement: Supplementary file 3 — CONSORT checklist. (PDF 301 kb) [file 13012_2018_841_MOESM3_ESM.pdf]

Additional file 3: CONSORT 2010 checklist of information to include when reporting a cluster randomised trial

Section/topic and item No	Standard checklist item	Extension for cluster designs	Section
**Title and abstract**			
1a	Identification as a randomised trial in the title	Identification as a cluster randomised trial in the title	Title
1b	Structured summary of trial design, methods, results, and conclusions (for specific guidance see CONSORT for abstracts)[11 12]	See table 2	Abstract
**Introduction**			
Background and objectives:			
2a	Scientific background and explanation of rationale	Rationale for using a cluster design	Introduction, Methods (Study design)
2b	Specific objectives or hypotheses	Whether objectives pertain to the cluster level, the individual participant level, or both	Introduction
**Methods**			
Trial design:			
3a	Description of trial design (such as parallel, factorial) including allocation ratio	Definition of cluster and description of how the design features apply to the clusters	Methods (Study design; Randomisation and allocation concealment)
3b	Important changes to methods after trial commencement (such as eligibility criteria), with reasons		Additional file 2
Participants:			
4a	Eligibility criteria for participants	Eligibility criteria for clusters	Methods (Recruitment of EDs and inclusion/exclusion criteria; Identification of patients and inclusion/exclusion criteria; and Recruitment of patients for follow-up and inclusion/exclusion criteria (NET-Plus only)).

Section/topic and item No	Standard checklist item	Extension for cluster designs	Section
4b	Settings and locations where the data were collected		Introduction
Interventions:			
5	The interventions for each group with sufficient details to allow replication, including how and when they were actually administered	Whether interventions pertain to the cluster level, the individual participant level, or both	Methods (Intervention/control); Additional file 5
Outcomes:			
6a	Completely defined prespecified primary and secondary outcome measures, including how and when they were assessed	Whether outcome measures pertain to the cluster level, the individual participant level, or both	Methods (Outcomes)
6b	Any changes to trial outcomes after the trial commenced, with reasons		Additional file 2
Sample size:			
7a	How sample size was determined	Method of calculation, number of clusters(s) (and whether equal or unequal cluster sizes are assumed), cluster size, a coefficient of intracluster correlation (ICC or *k*), and an indication of its uncertainty	Methods (Sample size)
7b	When applicable, explanation of any interim analyses and stopping guidelines		Not applicable
**Randomisation**			
Sequence generation:			
8a	Method used to generate the random allocation sequence		Methods (Randomisation and allocation concealment)
8b	Type of randomisation; details of any restriction (such as blocking and block size)	Details of stratification or matching if used	Methods (Randomisation and allocation concealment)
Allocation concealment			

Section/topic and item No	Standard checklist item	Extension for cluster designs	Section
mechanism:			
9	Mechanism used to implement the random allocation sequence (such as sequentially numbered containers), describing any steps taken to conceal the sequence until interventions were assigned	Specification that allocation was based on clusters rather than individuals and whether allocation concealment (if any) was at the cluster level, the individual participant level, or both	Methods (Randomisation and allocation concealment)
Implementation:			
10	Who generated the random allocation sequence, who enrolled participants, and who assigned participants to interventions	Replaced by 10a, 10b, and 10c	
10a		Who generated the random allocation sequence, who enrolled clusters, and who assigned clusters to interventions	Methods (Randomisation and allocation concealment)
10b		Mechanism by which individual participants were included in clusters for the purposes of the trial (such as complete enumeration, random sampling)	Methods (Identification of patients and inclusion/exclusion criteria; and Recruitment of patients for follow-up and inclusion/exclusion criteria (NET-Plus only)).
10c		From whom consent was sought (representatives of the cluster, or individual cluster members, or both) and whether consent was sought before or after randomisation	Methods (Recruitment of EDs and inclusion/exclusion criteria; Identification of patients and inclusion/exclusion criteria; and Recruitment of patients for follow-up and inclusion/exclusion criteria (NET-Plus only)).
Blinding:			
11a	If done, who was blinded after assignment to interventions (for example, participants, care providers, those assessing outcomes) and how		Methods (Blinding)
11b	If relevant, description of the similarity of interventions		Not applicable
Statistical methods:			
12a	Statistical methods used to compare groups for primary and	How clustering was taken into account	Methods (Effectiveness analyses)

Section/topic and item No	Standard checklist item	Extension for cluster designs	Section
	secondary outcomes		
12b	Methods for additional analyses, such as subgroup analyses and adjusted analyses		Additional file 2
**Results**			
Participant flow (a diagram is strongly recommended):			
13a	For each group, the numbers of participants who were randomly assigned, received intended treatment, and were analysed for the primary outcome	For each group, the numbers of clusters that were randomly assigned, received intended treatment, and were analysed for the primary outcome	Results (Figure 1)
13b	For each group, losses and exclusions after randomisation, together with reasons	For each group, losses and exclusions for both clusters and individual cluster members	Results (Figure 1)
Recruitment:			
14a	Dates defining the periods of recruitment and follow-up		Results (Recruitment of EDs and inclusion/exclusion criteria; Data collection processes)
14b	Why the trial ended or was stopped		
Baseline data:			
15	A table showing baseline demographic and clinical characteristics for each group	Baseline characteristics for the individual and cluster levels as applicable for each group	Results (Table 3 and Table 4)
Numbers analysed:			
16	For each group, number of participants (denominator) included in each analysis and whether the analysis was by original assigned groups	For each group, number of clusters included in each analysis	Results (Tables 5 and Table 6)
Outcomes and estimation:			
17a	For each primary and secondary outcome, results for each group, and the estimated effect size and its precision (such as 95% confidence interval)	Results at the individual or cluster level as applicable and a coefficient of intracluster correlation (ICC or *k*) for each primary outcome	Results (Table 5; Table 6; Intra-cluster correlations (ICCs) for the primary outcomes; Additional file 6)

Section/topic and item No	Standard checklist item	Extension for cluster designs	Section
17b	For binary outcomes, presentation of both absolute and relative effect sizes is recommended		Results (Table 5 and Table 6)
Ancillary analyses:			
18	Results of any other analyses performed, including subgroup analyses and adjusted analyses, distinguishing prespecified from exploratory		Results (Sensitivity analyses; Additional file 6)
Harms:			
19	All important harms or unintended effects in each group (for specific guidance see CONSORT for harms)		Not applicable
**Discussion**			
Limitations:			
20	Trial limitations, addressing sources of potential bias, imprecision, and, if relevant, multiplicity of analyses		Discussion (Study strengths and limitations)
Generalisability:			
21	Generalisability (external validity, applicability) of the trial findings	Generalisability to clusters and/or individual participants (as relevant)	Discussion (Study strengths and limitations)
Interpretation:			
22	Interpretation consistent with results, balancing benefits and harms, and considering other relevant evidence		Discussion
**Other information**			
Registration:			
23	Registration number and name of trial registry		Abstract; Methods; Additional file 1
Protocol:			
24	Where the full trial protocol can be accessed, if available		Additional file 1 is the full protocol
Funding:			
25	Sources of funding and other support (such as supply of drugs), role of funders		Declarations

*Page numbers optional depending on journal requirements.
