# Supplementary material for: Evaluation of a targeted, theory-informed implementation intervention designed to increase uptake of emergency management recommendations regarding adult patients with mild traumatic brain injury: results of the NET cluster randomised trial
Source: Implement Sci. 2019 Jan 17;14:4. doi: 10.1186/s13012-018-0841-7 (PMC6337860; doi:10.1186/s13012-018-0841-7)
Supplement: Supplementary file 4 — Patient identification protocol. (PDF 205 kb) [file 13012_2018_841_MOESM4_ESM.pdf]

Additional file 4: Patient identification protocol

STEP 1: Search experts (e.g. medical records managers or research officers at participating hospitals) created a sampling frame of potentially eligible patients, constructed from electronic searches of medical records using discharge diagnosis codes and triage free text terms indicative of mTBI. In hospitals unable to implement the search, the sampling frame consisted of all presentations in the two-months follow-up period. No patient identifying information was included in the sampling frames.

STEP 2: The records in the sampling frames were then randomly ordered and each record was assessed against the eligibility criteria by one auditor, using the available triage text and coding, until 100 potentially eligible patients were identified. Medical records of these patients were retrieved.

STEP 3: During site visits, trained, independent chart auditors determined the final eligibility of patients using the medical records. To ensure an even spread in presentation dates within each site's catchment period, the records were assessed in random order.

Step 1

Hospital

- Identify cohort (based on our search instructions*) and exclude by age
- Replace unit record (UR) numbers with consecutive numbers before sending report to Chart Auditor
- Save file with link between UR and consecutive numbers

→

Excluded

- In cases where search was not limited to 18 or older at time of presentation: exclude patients based on age

Step 2

Chart auditor

- Create RANDOM numbers and sort file on those
- Assessment based on sorted file. Exclude patients until 100 'potentially in'-patients identified (if available)
- Send file with 100 cases (including their consecutive numbers)

Hospital

- Link consecutive numbers with UR numbers and ask medical record department to retrieve those files

→

Included

- Impact to head
- Presented within 24 hrs after event
- Initial GCS 14 or 15

Excluded

- Penetrating injury
- Non-traumatic injury
- Left before treatment or discharged themselves

Step 3

Chart auditor visits hospital

- Assessment based on medical record data
- Data collected in order of random order list created by NET-Team

→

Included

- Impact to head
- Presented within 24 hrs after event
- Initial GCS 14 or 15

Excluded

- Penetrating injury
- Non-traumatic injury
- Left before treatment or discharged themselves
- Records missing

* Search instructions

In the report, please include all patients:

- who presented between [start date catchment period] and [end date catchment period]
- who are 18 years of age or older when they present
- who have a S0 code as discharge diagnosis from ED - either primary or secondary (so, include <u>full S0 range</u>)
- If you can search on free text in triage notes (e.g. presenting problem / nurse assessment text), then please <u>also</u> select patients who have the below free text words in Triage Notes
  Free text terms: = space

^ ASSAULT^
^ CHI ^
^ CHI,^
^ h/s ^
^ h/s,^
^ hs ^
^ hs,^
^ headstrike^
^ head ^
^ head,^
^ concuss^
^ LOC ^
^ LOC,^
