# Supplementary material for: Evaluation of a targeted, theory-informed implementation intervention designed to increase uptake of emergency management recommendations regarding adult patients with mild traumatic brain injury: results of the NET cluster randomised trial
Source: Implement Sci. 2019 Jan 17;14:4. doi: 10.1186/s13012-018-0841-7 (PMC6337860; doi:10.1186/s13012-018-0841-7)
Supplement: Supplementary file 5 — Overview of NET-Trial intervention and rationale for selection of components. (PDF 447 kb) [file 13012_2018_841_MOESM5_ESM.pdf]

## Additional file 5: overview NET Intervention including rationale for intervention components

| Component | Elements / techniques / additional information | Data source | Rationale: addressing or taking into account the following factors |
|---|---|---|---|
| **Multi-disciplinary Local Opinion Leader team** | * Recruitment of local opinion leaders (LOLs); one nursing and one medical in each hospital to lead the project and train staff | * Interviews – organisational factors<br>* EPOC review – local opinion leaders<br>* Practicalities | * Power & influence within professions rather than across<br>* Professions have their own systems to disseminate / implement changes<br>* Changes / leadership needs to be observable to keep momentum and give topic clout<br>* Leads are the most important way of 'integrating the recommendations into daily work'; being the "bridge" and "constant reminder" between "educational sessions" and "daily work processes" and ensuring changes will be sustained.<br>* Given the intensity needed and to cover staff on various shifts: need someone local |
| | * Description of 'ideal characteristics' of LOLs for ED Director | * Interviews – organisational factors<br>* Leadership theory | * Guide selection of LOLs: choose 'informal' leaders if appropriate |
| | * Nurse "main" lead | * Interviews – organisational factors | * Guideline-based intervention low compatibility with medical culture; good compatibility with nursing culture<br>* Use the stable forces |
| **Stakeholder Meeting** | Duration: 1 hour<br>Mode: face-to-face meeting with local stakeholders<br>Who "delivers": senior research team clinicians<br>Setting: in participating hospitals<br>Local stakeholders: prior to meeting discuss who local stakeholders are and organise they are in meeting | * Interviews – organisational factors | * Encourage organisational priority (acknowledging change is needed) / buy-in<br>* Start information provision / persuasive messages<br>* Ensure the 'key-people' are "on the same page" |
| | * Put this project in context Australian Commission on Safety and Quality in Healthcare requirements (standard 1)<br>* In the State of New South Wales a policy directive is in place | * Other documentation / information<br>* Interviews – organisational | * Show benefit at "organisational level" of project fitting with / contributing to meeting NSQHS requirements<br>* Intervention needs to be in line with policy directive<br>* Mixed perception regarding whether this project is in line with organisational priorities |
| | * Provide EDs with an opportunity to 'show case' leadership and commitment | * Interviews – organisational factors | * Strengthen organisational readiness / leadership & commitment<br>* Create buy-in from senior people involved in the implementation of the recommendations (organise top down, multi-disciplinary leadership) |
| | * Discuss recommendations & underlying evidence<br>For all three practices<br>* Information regarding behaviour & outcome / health consequences<br>* Persuasive messages<br>* Social processes of encouragement, pressure & support | * Interviews – organisational<br>* Interviews – TDF | * Strong evidence is pre-requisite for going through change effort<br>*Start of implementation of these techniques at senior clinician level, as in this setting generally implementation is top-down |
| | * Discuss how the recommendations fit with their workflow / processes & how eventually to will sustain these changes (Planning, implementation; Environmental changes).<br>* Focus is on the three separate practices (not one fixed protocol).<br>* Discuss anticipated local barriers and how to solve those | * Interviews – organisational factors | * Intervention needs to have potential for reinvention / fit with local practices / policy directives; needs to be worked into something that fits with their practices<br>* Being subspecialty at the entry-point of the hospital means many specialties have requests with respect to the management if they were to admit patients under their care<br>* Absence of agreed cross-unit pathways / protocols<br>* Agreement between different specialties generally difficult to organise |
| **Train the Trainer Day** | 1 day event (8 hours) – delivered twice in two different capital cities<br>Attended by clinical leads<br>Setting: off site conference venue<br>Delivered by senior research team clinicians / clinical opinion leaders | * Interviews – organisational factors<br>* Cochrane EPOC reviews<br>* Follows from TTT model | * Someone needed to train 'the rest of the troops'<br>* LOLs need to have the clinical + leadership knowledge and skills in order to provide the local education / training<br>* LOLs need to know and understand the importance of their role<br>* LOLs need to receive all materials and resources needed in the local training<br>* LOLs need to understand what is expected from them in terms of intervention delivery and fidelity assessment |
| *1. Introduction to the day* | Set the scene: NET Program and KT | | * Set the scene / KT capacity building |
| *2. Teach content local workshops (clinical focus)* | Teach leads what they will teach staff (clinical)<br>Teach all participants together (nursing & medical) | * Interviews – organisational factors | * Inter-professional boundaries; power and influence within groups rather than across. This is often seen as a barrier to safe team based care<br>* Care is team-based (who's doing what depends also on who's around etc) |

| Component | Elements / techniques / additional information | Data source | Rationale: addressing or taking into account the following factors |
|---|---|---|---|
| | | | * Hospitals vary a lot in terms of their staff available so LOLs should ideally function as a team. |
| *Clinical practice: PTA* | | | TDF domains in **bold** |
| **Domains to address (in order of perceived importance):**<br>**1.Knowledge**<br>**2.Beliefs about consequences**<br>**3.Environmental context and resources**<br>**4.Beliefs about capabilities**<br>**5. Skills**<br>**6.Social professional role and identity**<br><br>Note: in the skills demonstration modelling and rehearsal the 3 practices were combined; however for purposes of this overview they are included in all three clinical practices | * Information provision on clinical practice and outcomes: what PTA is, difference with GCS and why it is important<br>* Health consequences<br>* Pros and cons | * Interviews - TDF | * Little knowledge on what PTA is, how it differs to GCS and the benefits of doing a formal assessment **[knowledge]**<br>* **Beliefs about consequences** |
| | * Information provision on existence validated tools and its content | * Interviews – TDF | * Little / no awareness of existence tools **[knowledge]** |
| | * Provide information on how & when (e.g. in what patients) to do the assessment / use the tool<br>* Antecedents (information on environmental situations; events that predict when PTA tool would be or wouldn't be used) | * Interviews – TDF | * Anticipated lack of **skills**<br>* Little **knowledge** of how / when to do assessment |
| | * Persuasive messages by respected opinion leaders / influential peers (both presenters and facilitators) , including nurses already screening for PTA<br>* Social processes by having the influential people to deliver messages (regarding importance of doing structured assessment / not discharging patients who are still in PTA as well as targeting beliefs that this behaviour is outside the ED realm | * Interviews – TDF<br>* Interviews – organisational factors | * Positive **beliefs about consequences** regarding more 'objective' assessment (Confirmation of enabler in nurses)<br>* Not convinced about the added benefit of using a PTA tool (**beliefs about consequences**): doctors<br>* **Social professional role and identity**<br>* Relatively low tension for change for managing acute symptoms |
| | * Persuasive messages with bedside experiences (e.g. time it takes to complete assessment; actually saves time at ward etc.) | *Interviews - TDF | * **Beliefs about consequences** |
| | * Skills demonstration / modelling<br>* Provide goal/target for the specified behaviour or outcome | *Interviews - TDF | *Anticipated lack of **skills** |
| | * Rehearsal of skills: role play (in pairs - using vignettes: simulated patients)<br>* Graded task i.e. start with basic vignette and then progress to more difficult cases | * Interviews – TDF | * Anticipated lack of **skills / beliefs about capabilities**<br>* Little **knowledge** of how / when to do assessment → practice doing these in the same manner all the time |
| | * Teach LOLs on what materials they will be receiving and working with: PTA-tool (intranet and hard-copy)<br>* Prompts, triggers, cues (physical reminders e.g. stickers, posters etc.) | * Interviews – TDF | * **Environmental context & resources** |
| *Clinical practice: CT* | | | TDF domains in **bold** |
| **Domains to address (in order of perceived importance):**<br>**1.Beliefs about consequences**<br>**2.Environmental context and resources**<br>**3.Beliefs about capabilities**<br>**4.Social influences**<br>**5.Knowledge**<br>**6. Behavioural regulation**<br>**7. Memory, attention & decision processes** | * Information provision on clinical practice and outcome (need to do risk assessment / radiation / frequency of CT evident lesions / trade-off etc.)<br>* Health consequences | * Interviews - TDF | * Lack of **Knowledge/ awareness** around radiation involved / figures re CT evident lesions<br>* **Beliefs about consequences** |
| | * Information/education on available head scanning rules, how they are developed, differences between them | * Interviews - TDF | * **Knowledge** (junior doctors not always aware of validated rules)<br>* **Beliefs about consequences** |
| | * Information on how and when to use a rule in decision making and explaining how rule should be used "to the rule" (due to specificity/sensitivity)<br>* Antecedents (information on environmental situations; events that predict when rules are and are not used) | * Interviews - TDF | * **Knowledge** |
| | * Persuasive messages by respected medical opinion leaders / influential peers (both presenters and facilitators) , including information on life-time cancer risk from radiation<br>* Social processes | * Interviews – TDF<br>* Interviews – organisational factors | ***Social influences**<br>* **Beliefs about consequences**<br>*Particularly in medical clinicians: power & influence within professional silo's |
| | * Skills demonstration / modelling (how to correctly use a rule)<br>* Provide goal/target for the specified behaviour or outcome | * Interviews - TDF | * **Beliefs about capabilities**<br>***Knowledge** |
| | * Rehearsal: role play (in pairs - using vignettes: simulated patients)<br>* Graded task i.e. start with basic vignette and then progress to more | * Interviews - TDF | * **Beliefs about capabilities** |

| Component | Elements / techniques / additional information | Data source | Rationale: addressing or taking into account the following factors |
|---|---|---|---|
| | difficult cases (e.g. practicing difficult situations like parents who demand a CT scan) | | |
| | * Teach LOLs on what materials they will be receiving and working with e.g. rules in various formats such as lanyards with CT rule attached to it)<br>* Prompts, triggers, cues (physical reminders e.g.  stickers, posters etc. | * Interviews – TDF | * **Environmental context & resources**<br>* **Memory, attention & decision**<br>* **Behavioural regulation** |
| ***Clinical practice: INFO*** | | | TDF domains in **bold** |
| **Domains to address (in order of perceived importance):**<br>**1.Beliefs about consequences**<br>**2.Environmental context and resources**<br>**3. Memory, attention & decision processes**<br>**4.Social/professional role and identity**<br>**5.Knowledge** | * Information provision on behaviour and outcome (explain key content - difference between our booklet and others)<br>* Discuss health consequences of not providing reassuring information | * Interviews – TDF<br>* Interviews – organisational factors | * **Knowledge**<br>* Reinforce positive **beliefs about consequences**<br>* Strengthen readiness for change for longer-term management (prevent patients 'falling through the cracks') |
| | * Persuasive messages on outcomes (e.g. using patient case)<br>* Social processes of encouragement | * Interviews – TDF | * **Beliefs about consequences** |
| | * Information on team-behaviours: role clarity | * Interviews – TDF | * **Social professional role and identity** |
| | * Skills demonstration / modelling (how to correctly discuss the information in the booklet with a patient verbally)<br>* Provide goal/target for the specified behaviour or outcome | | * Included as part of skills training for appropriately managing mTBI patients. Although participants did not voice doubts regarding their skills in this area – we anticipated staff would benefit from knowing which elements of the patient information are particularly important to address verbally and practice this. |
| | * Rehearsal: role play (in pairs - using vignettes: simulated patients) | | |
| | *Problem solving (discuss potential barriers and how to deal with those – in pairs)<br>* Planning, implementation (e.g. we all know how to hand out a booklet; now how do we ensure we actually do this) | * Interviews – TDF | * **Memory, attention and decision processes** |
| | * Teach LOLs on what materials they will be receiving and working with , e.g. electronic and printed booklets in various languages)<br>* Offer to print stickers with contact details of relevant local services (e.g. Acquired Brain Injury clinic) to stick in booklet<br>* Prompts, triggers, cues (physical reminders e.g. stickers, posters etc.) | * Interviews – TDF | * **Environmental context & resources**<br>* **Memory, attention & decision processes**<br>*Strengthen readiness for change for longer-term management (prevent patients 'falling through the cracks') |
| *3. Leadership training* | | | *Empower LOLs to be effective trainers |
| | * Provide information on their role as LOL (being the bridge between education sessions and daily practice)<br>* Provide information on the importance of blame-free organisational cultures / organisational learning | * Interviews – organisational factors<br>* EPOC reviews | * LOLs needs to have insight into their role and the importance of it (being the constant reminder between the education and applying in practice; source of support & encouragement; be enthusiastic, engage people)<br>* General lack of feedback in ED<br>* Impressions of existence of "punitive culture" in ED |
| | * Provide information on the materials they will receive to conduct the staff training | | * Materials have been prepared by the research team, including key messages, scripts etc. so as to ensure |
| | Time for the teams to discuss with their local LOL partner how to do the local training using structured set of questions, including thinking through (common) barriers and solutions and strategies to sustain the changes<br>* Action planning<br>* Problem solving<br>* Planning, implementation<br>* Goal/targeted behaviour (e.g. how many staff will be trained)<br>* Modelling (nurse talking to her experiences) | | * Planning for sustainability |
| | * Instruct LOLs on fidelity measurement requirements | | * Research tasks |
| | * Help available  – communication with research team | | * Provide support |
| *4. closing to the day* | | | |
| | | | |
| **Local workshops** | Mode of delivery: slides for face-to-face presentations (PTA, CT, INFO) and materials for face to face skills demonstration session + pre-recorded versions of all training sessions for staff who could not attend face-to-face sessions | * Interviews – organisational factors<br>* Cochrane EPOC reviews<br>* Follows from TTT model | |

| Component | Elements / techniques / additional information | Data source | Rationale: addressing or taking into account the following factors |
|---|---|---|---|
| | Setting: participating hospitals<br>Delivered by: LOLs in each site<br>Attended by staff in each ED<br>Duration: sessions designed to fit in (less than) 20 minutes | | |
| | * Opportunity to bring in local influential clinical experts to co-deliver info/training? | * Interviews – organisational factors | *Power & influence |
| | * Design brief sessions so that they can use them as little building blocks (either combine or separate) | * Interviews – organisational factors | *High turn-over rates<br>* Interventions needs to fit with local change management practices<br>* Little organisational slack |
| | PTA session: content elements is similar to TTT | *Interviews TDF | See TTT |
| | CT session: content elements is similar to TTT | | See TTT |
| | INFO session: content elements is similar to TTT | | See TTT |
| | Skills training : demonstration session by LOL to staff on optimal management (rehearsal of relevant skills by staff is optional) | | See TTT |
| | * Verbal reminders / feedback / support from LOLs to staff | | * Flowing from LOL model: they are the "supportive bridge" between education sessions and daily care |
| | | | |
| **Provision of tools and materials** | * Environmental changes: provide sites with tools and materials needed:<br>-A-WPTAS (electronic + paper)<br>-CT ITIM rules (+ links to other rules)<br>-Info-booklet (English, Greek, Italian, Arabic, Chinese & Vietnamese) | *Interviews TDF | * Environmental context & resources<br>* Memory & attention processes |
| | *Decide "fixed content" = clinical; allow changes to logo's telephone numbers etc. | * Interviews – organisational factors | *Intervention needs to be in line with local processes / need for "re-invention" |
| | * General reminding / promotional materials (e.g. NET pens) | | |
| | | | |
| **Data collection Reminder (both intervention & control group)** | *General reminder to optimise data collection processes for mTBI patient group | | |
| | | | |
| **ITIM guideline (both intervention & control group)** | * Electronic copy of the guideline | | |
| **Additional components** | | | |
| **MoU or legal agreement** | * Formalise participation, 'contract' signed by appropriate senior management (e.g. hospital CEO) | * Interviews – organisational factors<br>* Research Governance | * Create "organisational (vertical) leadership"<br>* Comply with organisational governance processes |
| | | | |
| **Reimbursement** | * Provide lump-sum honorarium for services rendered | * Interviews – organisational factors | * Very little organisational slack |
| | | | |
| **Endorsement** letters from relevant ED Colleges (both medical and nursing) | * Social influence from professional bodies | * Interviews – organisational factors<br>* Theory around professionalism & social influence | * Influence & power organised by / within separate professions |
