# Supplementary material for: Evaluation of a targeted, theory-informed implementation intervention designed to increase uptake of emergency management recommendations regarding adult patients with mild traumatic brain injury: results of the NET cluster randomised trial
Source: Implement Sci. 2019 Jan 17;14:4. doi: 10.1186/s13012-018-0841-7 (PMC6337860; doi:10.1186/s13012-018-0841-7)
Supplement: Supplementary file 6 — Extra tables. ICCs and estimated effects of the intervention on clinical practice and patient outcomes, adjusting for the minimisation criteria only. (PDF 303 kb) [file 13012_2018_841_MOESM6_ESM.pdf]

**Additional file 6: Extra Tables.** ICCs and estimated effects of the intervention on clinical practice and patient outcomes, adjusting for the minimisation criteria only.

Estimated effects of the intervention on clinical practice outcomes – minimisation criteria only

| | NET Control[1] | | NET Intervention[2] | | | | |
|---|---|---|---|---|---|---|---|
| | **No. patients** | **No. (%)** | **No. patients** | **No. (%)** | **Adj. ORs** | **95% CI** | **p-value** |
| Appropriate post-traumatic amnesia screening (PTA)* | 1050 | 12 (1.1) | 893 | 117 (13) | 15.6 | (5.0, 48.8) | < 0.001 |
| PTA screening-tool | 1050 | 15 (1.4) | 893 | 152 (17) | 20.2 | (6.9, 59.4) | < 0.001 |
| Memory - clinical assessment | 1050 | 272 (26) | 893 | 303 (34) | 1.6 | (1.2, 2.1) | 0.001 |
| CT scan-clinical criteria (CT)§ | 494 | 337 (68) | 491 | 352 (72) | 1.2 | (0.8, 1.6) | 0.349 |
| CT-scan (all) §§ | 1050 | 458 (44) | 893 | 446 (50) | 1.3 | (1.0, 1.7) | 0.046 |
| Provision of written patient information (INFO) | 944 | 175 (19) | 785 | 160 (20) | 1.2 | (0.8, 1.7) | 0.367 |
| Safe discharge based on PTA and INFO | 944 | 2 (0.2) | 785 | 45 (6) | 28.8 | (6.1, 128.9) | < 0.001 |
| Safe discharge based on PTA, CT, and INFO | 413 | 0 (0) | 402 | 14 (3.5) | 1.8§§§ | (1.1, 3.0) | 0.022 |

1 number of clusters = 17
2 number of clusters = 14
ORs = odds ratios
* Primary outcome
§ Criteria that justify a scan are: age 65 or older; GCS<15; amnesia; suspected skull fracture; vomiting and coagulopathy. Only the subset of patients who have these symptoms noted in the medical records are included in the analysis.
§§ This outcome was additional to the ones specified in the trial protocol (Additional file 2)
§§§ For this outcome, because there were no safe discharges in the control group, a cluster-level analysis was undertaken resulting in a ratio of geometric mean proportions. Details available in Additional file 2.

Estimated effects of the intervention on patient outcomes – minimisation criteria only

| | | NET-Plus Control | | NET-Plus Intervention | | | | |
|---|---|---|---|---|---|---|---|---|
| ***Patient interview responses*** | **Value range** | **No. patients / clusters** | **Mean (sd) / N (%)** | **No. patients / clusters** | **Mean (sd) / N (%)** | **Adjusted Effect^** | **95% CI** | **p-value** |
| Anxiety[1] | 0 to 21 | 218 / 14 | 4.3 (4.01) | 125 / 10 | 3.4 (3.58) | MD -0.61^^ | (-1.46, 0.24) | 0.158 |
| Post-concussion symptoms (RPQ-13)[2] | 0 to 52 | 218 / 14 | 6.7 (8.65) | 125 / 10 | 4.7 (5.52) | MD -1.26^^ | (-2.88, 0.36) | 0.127 |
| Post-concussion symptoms (RPQ-3)[3] | 0 to 12 | 218 / 14 | 1.2 (1.83) | 125 / 10 | 0.90 (1.44) | MD -1.10^^ | (-0.53, 0.19) | 0.352 |
| Not returned to normal activities[4] | 0 or 1 | 218 / 14 | 41 (19%) | 126 / 10 | 16 (13%) | OR 0.67^^^ | (0.28, 1.52) | 0.326 |
| SF6D HRQoL[5] | 0.35 to 1 | 208 / 14 | 0.78 (0.14) | 123 / 10 | 0.80 (0.13) | MD 0.03^^ | (0.00, 0.06) | 0.031 |
| mTBI-related re-presentation[6] | 0 or 1 | 1050 / 17 | 25 (2.4%) | 893 / 14 | 39 (4.4%) | OR 1.92^^^^ | (1.07, 3.37) | 0.029 |

1 Anxiety measured using the anxiety items in the Hospital Anxiety and Depression Scale giving a score between 0 and 21, higher scores indicate higher levels of anxiety and a score > 7 indicates clinically significant anxiety.
2 Post-concussion symptoms measured using the 13 item Rivermead scale (RPQ-13) giving a score between 0 and 52, higher scores indicate greater severity of post-concussion symptoms.
3 Post-concussion symptoms measured using the 3 item Rivermead scale (RPQ-3) giving a score between 0 and 12, higher scores indicate greater severity of post-concussion symptoms.
4 Whether or not a patient returned to normal activities was indicated by the patient answering no to any of the following: "Are you doing the same working hours as before the incident?" "Are you studying the same hours as before the incident?" "Are you back to (your) other normal activities such as gardening, buying groceries, visiting friends or family, or other leisure activities etc.?"
5 SF6D index scores, derived from SF12v2 raw data using weights from Brazier & Roberts [1].
6 Chart audit data.
^ Adjusted effects from models fitted using generalised estimating equations with an exchangeable correlation structure (unless otherwise

noted) and robust variance estimation to allow for clustering within hospitals. Models adjusted for the design strata and pre-specified confounders (see 'Effectiveness Analyses' section ). Adjusted effects are adjusted mean differences (denoted MD) or adjusted odds ratios (denoted OR).

^^ Modelled with independent within-group correlation structure. Details available in Additional file 2.

## Intra-cluster correlations (ICCs) for clinical practice outcomes

| | Combined | | Control | | Intervention | |
|---|---|---|---|---|---|---|
| | ICC** | 95% CI** | ICC** | 95% CI** | ICC** | 95% CI** |
| Appropriate post-traumatic amnesia screening (PTA)* | 0.12 | (0.06, 0.19) | 0.20 | (0.08, 0.32) | 0.06 | (0, 0.11) |
| PTA screening-tool | 0.15 | (0.08, 0.22) | 0.19 | (0.07, 0.30) | 0.07 | (0.01, 0.13) |
| Memory - clinical assessment | 0.05 | (0.02, 0.09) | 0.07 | (0.01, 0.13) | 0.03 | (0, 0.06) |
| CT scan-clinical criteria (CT)§ | 0.05 | (0.01, 0.09) | 0.03 | (0, 0.07) | 0.07 | (0, 0.14) |
| CT scan (all) §§ | | | | | | |
| Provision of patient information (INFO) | 0.04 | (0.01, 0.06) | 0.02 | (0, 0.04) | 0.06 | (0, 0.12) |
| Safe discharge based on PTA and INFO | 0.06 | (0.02, 0.10) | 0 | (0, 0.02) | 0.04 | (0, 0.08) |
| Safe discharge based on PTA, CT, and INFO | 0.03 | (0, 0.07) | *** | | 0.02 | (0, 0.06) |

* Primary outcome

§ Criteria that justify a scan are: age 65 or older; GCS<15; amnesia; suspected skull fracture; vomiting and coagulopathy. Only the subset of patients who have these symptoms noted in the medical records are included in the analysis.

§§ This outcome was additional to the ones specified in the trial protocol

** ICC point estimates are calculated from ANOVA. Confidence intervals for the ICCs were bootstrapped using the combination of bootstrap and loneway commands in Stata (StataCorp. 2015. Stata Statistical Software: Release 14. College Station, TX: StataCorp LP). We allowed clustering of observations within EDs. Bias corrected 95% confidence intervals were calculated using 1000 replicates.

*** Could not calculate the ICC or its confidence interval due to no observed events.

## Intra-cluster correlations (ICCs) for patient outcomes

| | Combined | | Control | | Intervention | |
|---|---|---|---|---|---|---|
| | ICC** | 95% CI** | ICC** | 95% CI** | ICC** | 95% CI** |
| Anxiety[1] | 0.02 | (0.01, 0.07) | 0 | (0,0.07) | 0.09 | (0, 0.26) |
| Post-concussion symptoms (RPQ-13)[2] | 0.04 | (0.01, 0.11) | 0.02 | (0, 0.07) | 0.09 | (0, 0.24) |
| Post-concussion symptoms (RPQ-3)[3] | 0.02 | (0, 0.10) | 0.02 | (0, 0.08) | 0.03 | (0, 0.20) |
| Not returned to normal activities[4] | 0.04 | (0.03, 0.10) | 0.01 | (0, 0.08) | 0.11 | (0.02, 0.31) |
| SF6D HRQoL[5] | 0.02 | (0, 0.08) | 0 | (0, 0.08) | 0.06 | (0, 0.17) |
| mTBI-related re-presentation[6] | 0 | (0, 0.01) | 0.01 | (0, 0.02) | 0 | (0, 0.02) |

[1] Anxiety measured using the anxiety items in the Hospital Anxiety and Depression Scale giving a score between 0 and 21, higher scores indicate higher levels of anxiety and a score > 7 indicates clinically significant anxiety.

[2] Post-concussion symptoms measured using the 13 item Rivermead scale (RPQ-13) giving a score between 0 and 52, higher scores indicate greater severity of post-concussion symptoms.

[3] Post-concussion symptoms measured using the 3 item Rivermead scale (RPQ-3) giving a score between 0 and 12, higher scores indicate greater severity of post-concussion symptoms.

[4] Whether or not a patient returned to normal activities was indicated by the patient answering no to any of the following: "Are you doing the same working hours as before the incident?" "Are you studying the same hours as before the incident?" "Are you back to (your) other normal activities such as gardening, buying groceries, visiting friends or family, or other leisure activities etc.?"

[5] SF6D index scores, derived from SF12v2 raw data using weights from Brazier & Roberts [1].

[6] Chart audit data.

1. Brazier JE, Roberts J. The estimation of a preference-based measure of health from the SF-12. Med Care 2004, 42(9):851-859.
